# Financial inclusion helps rural households address climate risk

**DOI:** 10.1038/s41598-023-34844-y

**Published:** 2023-05-16

**Authors:** Ashwini Chhatre, Prachi Deuskar, Javed Mohib, Deepanshi Bhardwaj

**Affiliations:** 1 Indian School of Business, Gachibowli, Hyderabad, 500032 India; 2 Tarneit, Australia; 3 UCL School of Management, University College London, London, E14 5AB UK

**Keywords:** Climate-change adaptation, Climate-change policy

## Abstract

Financial inclusion plays an important role in helping households manage risks, but its role in mitigating climate risks is unexplored. Access to formal financial institutions in regions with high climate risks increases households’ access to liquidity that they need to buffer against climate shocks. Using longitudinal data from 1082 rural households located in the semi-arid tropics in India, we find that households facing high climate risks hold a higher proportion of assets in liquid form. Access to formal financial services, however, reduces the need to keep liquid assets to be able to respond to high climate variability. Our results suggest that expanded financial inclusion in regions with high climate variability can reallocate resources held in unproductive liquid assets to invest in climate adaptation.

## Introduction

As it becomes increasingly clear that some degree of catastrophic climate change is unavoidable, the world is gearing up to manage climate change by enabling adaptation at all levels^1–3^, with a focus on rural households in low- and middle-income countries as one of the most vulnerable social groups^4^. Scholars have proposed multiple approaches that include reducing vulnerability through livelihood diversification^5–7^, increasing adaptive capacity by improving governance^8^, and using technology to provide advanced and early warning of impending extreme weather events^9^. Until now, these proposals have mostly been translated into discrete projects, implemented with the support of multilateral institutions, philanthropic foundations, and international organizations^10,11^. At the same time, it is apparent that information about the potential impacts of climate change derived from downscaled climate models will not be available at sufficiently high resolution or precision for planning effective climate adaptation at the household or community level^12^. To the extent that our current state of knowledge about climate change is restricted to probabilistic estimates of changes in climate risk in the medium to distant future, the emerging focus on adaptation to unavoidable and inevitable climate change is welcome.

The state of our knowledge about rural households in semi-arid tropics is worse than our knowledge of potential climate impacts on these households. We know that rainfed farmers are directly at risk from unpredictable weather events such as delayed monsoon onset, long dry spells, unseasonal frost or hail, and in the extreme, droughts and floods^4,13^. An important strand in scholarship on climate adaptation examines the strategies, such as occupational diversification, seasonal migration, and reliance on social networks, employed by households themselves to manage risks^5,14–16^. One important aspect of such strategies often addressed in passing in this literature is the common practice of holding a fraction of assets in liquid form to provide quick access to cash in times of negative income shocks^17–19^. Due to the nature of imperfect markets in rural areas, households face a steep trade-off between liquidity and returns in the limited avenues available to them, with disproportionately low returns on distress sale of assets. Such coping strategies will put households on a lower-growth trajectory in the long-term by lowering ex-ante investment in productive capital^17^. We expect households living in regions with high climate variability to hold a higher proportion of their assets in a form more amenable to quick liquidation, in addition to other risk mitigation strategies. However, holding liquid assets is not costless as it reduces resources available for productive investment or consumption.

We estimate the impact of access to formal financial institutions on the ability of rural households to address climate risks. In the absence of liquid assets, rural households turn in the last resort to consumption loans from informal sources and often at exorbitant interest rates. Such predatory lending practices exacerbate household vulnerability and prolong recovery from climate shocks. In response to high and increasing levels of indebtedness, governments, bilateral and multilateral organizations, and philanthropic foundations have focused attention to expanding financial inclusion of the poor through multiple mechanisms. Indeed, there is evidence that financial inclusion helps people manage risks better^20^, enabling them to make more productive investments^21,22^. Financial inclusion enables more efficient allocation of scarce resources towards addressing climate risk. Rural households with access to formal financial institutions need to hold fewer assets in liquid form, compared to those without such access^19,23^, making resources available for investments in adaptation to climate change.

We examine the extent of assets held in liquid form as a hedge against climate risk, with and without financial inclusion. Our analysis draws on granular household-level panel data of 1082 households, tracked across 2010–2014 (SI Materials and Methods). The data are obtained from International Crop Research Institute for the Semi-Arid Tropics—Village Dynamics in South Asia (ICRISAT-VDSA) and cover households in 30 villages across nine states in Indian semi-arid tropics^24^. The dataset includes household characteristics such as size, caste, landholdings, assets, liabilities, financial transactions as well as their exposure to different income shocks and coping mechanisms. We examine liquid assets—defined as cash, gold, silver, and financial investments—as a percentage of the monetary value of total household assets.

Arguably, financial inclusion is a multidimensional concept. Indeed, Camara and Tuesta^25^ consider the dimensions of access, usage, and barriers to inclusion and Avom et al.^26^ further add the dimension of penetration while constructing country-level financial inclusion indices. Using our granular data, we capture financial inclusion of the *household* through two indicators: 1. whether the household saves in or borrows from banks or other formal financial intermediaries (HH_BANKED); 2. if the household reports ‘bank’ as being in their top three reliable sources of support in case of a climate shock (BANK_TOP3). HH_BANKED reflects access as well as penetration of formal financial institutions. BANK_TOP3, in addition to these two dimensions, also indicates usage and low barriers since the household considers banks as a reliable source. Globally, financial inclusion is dominated by access to financial institutions like banks with 60% of the adult population having an account with a financial institution and only 2% having a mobile money account^11^.

However, one might wonder whether banks are the right mode to measure financial inclusion of the rural population. This concern is assuaged by expansion of rural baking in the Indian context. Since 1969, with the beginning of the social banking phase, India has made a conscious effort through various policy choices to expand rural banking^27–29^ among others. As a result, as Garg et al.^30^ note, bank access in rural areas has improved consistently over time with the average distance of unbanked villages to the nearest banked centre coming down from more than 20 km in 1969 to less than 10 km by 2010. India’s central bank implements the Priority Sector Lending policy, which stipulates that 18% of Net Adjusted Bank Credit must be delivered to the Agriculture sector, with slightly less than half of this dedicated for small and marginal farmers. This support, however, can only be delivered through banks in rural areas. Therefore, expanding financial inclusion in rural areas requires bank accounts for delivery of subsidized credit and other financial products. Over the last ten years, this policy has received further impetus through expanded investment in creating demand for zero-balance savings accounts by expansion of Direct Cash Transfer of subsidies and credit as a statutory requirement for receiving public welfare benefits. Savings products help by providing safety from theft, helping curb impulse spending, and facilitating better cash management^20^. Narayanan^31^ finds that higher agricultural credit is associated with increase in use of fertilizers and pesticides and higher investment in tractors. Young^32^ finds that expansion of rural banking results in increase in total output for several important crops.

**Figure 1:**
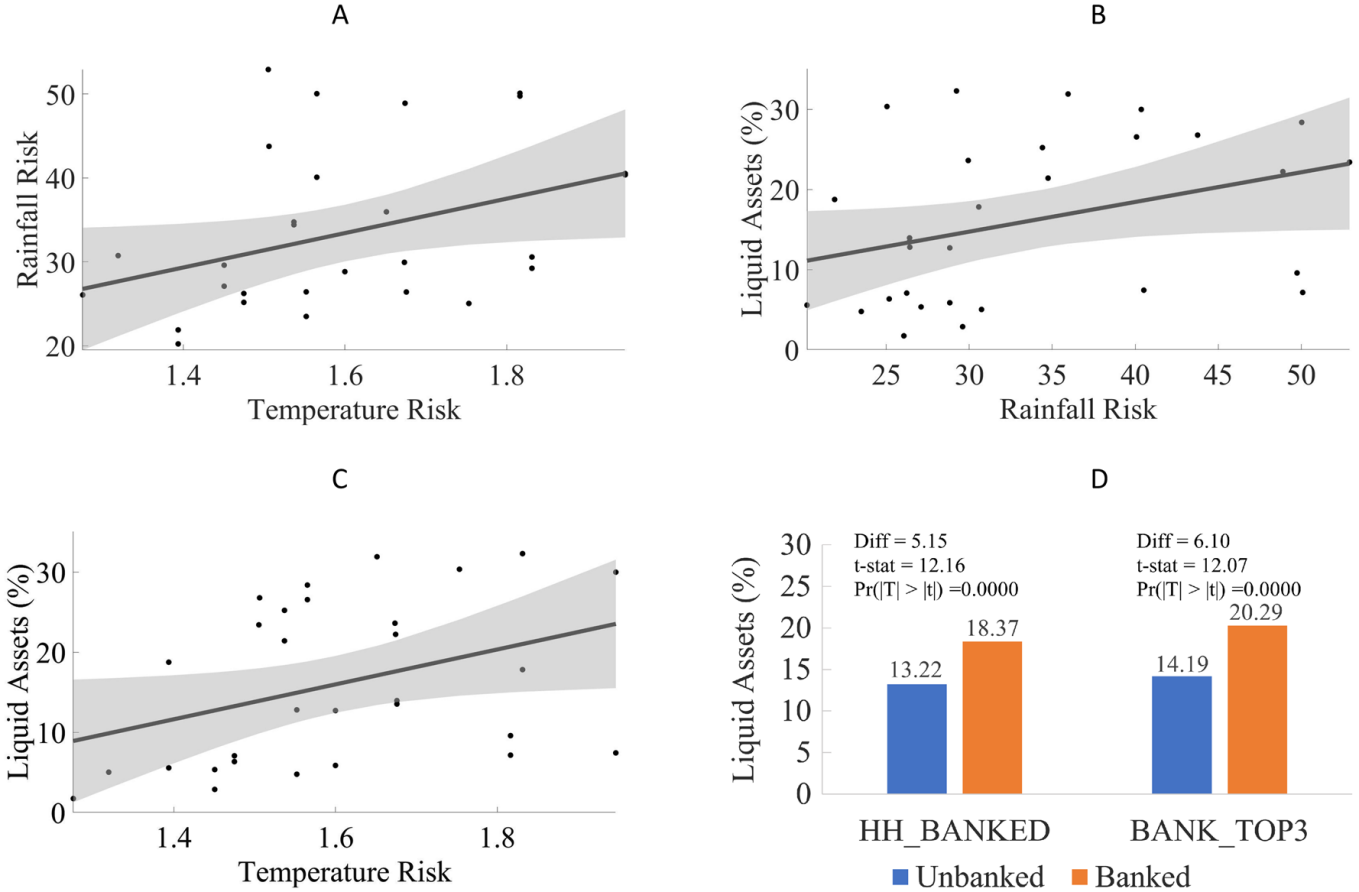
The figure shows the relationship between climate risk, liquid assets, and financial inclusion. (**A**) Rainfall Risk in a village on Y-axis against Temperature Risk on the X-axis. (**B**) Average percentage of liquid assets in a village on Y-axis against Rainfall Risk on the X-axis. (**C**) Average percentage of liquid assets in a village on Y-axis against Temperature Risk on the X-axis. (**D**) Average percentage of liquid assets for banked and unbanked households, along with the results of a two-sample t-test for the difference. Panels A, B, and C also show the fitted line with 95% confidence interval. Rainfall Risk and Temperature Risk are measured as the coefficient of variation of annual rainfall and mean temperature from 1951 to 2014. Yearly values are total rainfall and mean of daily average temperature, during 120 days after monsoon onset. Banked households are defined as either (1) those who save or borrow from banks or other formal financial intermediaries (HH_BANKED); or (2) those who report ‘bank’ as being in their top three reliable sources of support in case of a climate shock (BANK_TOP3).

We use coefficient of variation in long-term rainfall and temperature at the village level as indicators of climate risk, derived using data from India Meteorological Department covering 1951–2014 (Tables S1, S2). These two dimensions of risk show a moderate positive correlation (Pearson’s Rho = 0.37, *p* = 0.043) but with high independent variation (Fig. 1A).

Typical of rural populations in the semi-arid tropics, 59% of the households in our sample experienced climate shocks in at least one of 5 years and 13% faced them in more than 2 years (Fig. S1). Out of the 633 households that faced at least one adverse climate shock affecting their livelihoods, a large fraction (358, 57%) reported their own savings as the primary coping mechanism (Fig. S2). As a consequence, the proportion of liquid assets held by rural households increase in proportion to rising climate variability (Fig. 1B,C). This is consistent with our proposition that households set aside a larger liquidity buffer to mitigate risks arising from high climate variability. For households facing a climate shock, their most reliable external source of assistance is kin and relatives followed by friends, with banks coming in fifth behind village community and money lender (Fig. S3). When the households have bank in their top 3 reliable sources of assistance, they rely less on moneylenders. In our sample, 20.1% of households report moneylender being in the top 3 sources as opposed to 29.8% households when bank is not in top 3 (Two-sample t-test; t-stat = 6.64, Pr(|T| >|t|) = 0.000, Fig S4). Households with access to formal financial institutions keep a slightly larger share of their assets in liquid form (Liquidity Ratio = 20.29% for households reporting Bank in their top 3 reliable sources of assistance in a crisis, compared to 14.19% for households that do not. Two-sample t-test; t-stat = 12.07, Pr(|T| >|t|) = 0.0000, Fig. 1D). This positive relationship could be driven by the selection that households who want to save more or have a greater ability to save are more likely to rely on banks. We address this endogeneity through instrumental variable approach as discussed next.

How does the portfolio of banked households compare to that of the unbanked in relation to climate risk? To understand the relationship between climate risk, financial inclusion, and household liquidity, we model the fraction of liquid assets as a function of the climate risk variables, a financial inclusion indicator, and its interaction with climate risk. We appreciate that whether the household is financially included depends on many observed and unobserved factors, some of whom may be correlated with the fraction of assets held in liquid form. To address this issue, we use instrumental variables regression with village indicators as instruments for financial inclusion, while controlling for relevant household and village level variables in the main model (SI Materials and Methods).

**Figure 2:**
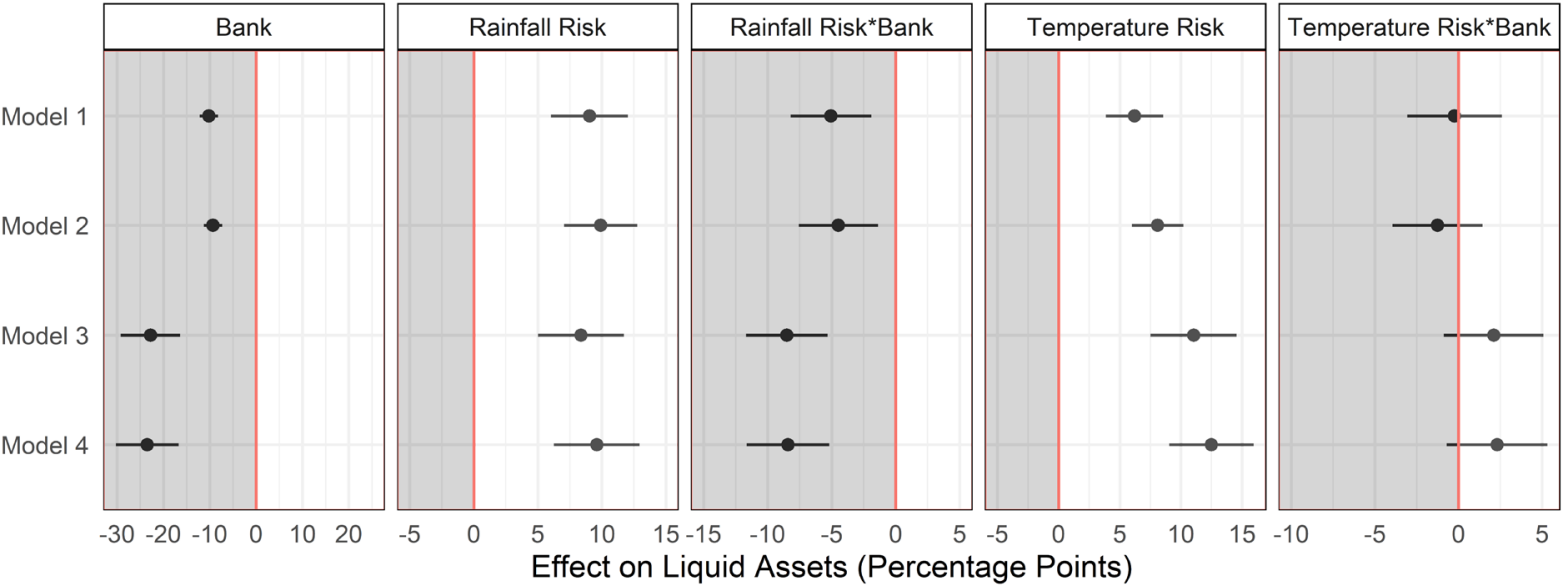
This figure shows the marginal effects and 95% confidence intervals from an instrumental variable regression of fraction of liquid assets on an indicator of financial inclusion (Bank), Rainfall Risk, Temperature Risk, and their interaction with Bank from four models. The dependent variable is the inverse normal transformation of the fraction of liquid assets. Black circles show the marginal effect on the percentage of liquid assets. Black lines indicate the 95% confidence intervals. Rainfall Risk and Temperature Risk are measured at the village level and are standardized to have mean 0 and unit standard deviation across villages. All the models control for household size, village altitude (meters above mean sea level), village nightlights (as a proxy for level of economic activity), and district, caste and farmer class fixed effects. Models 1 and 2 use BANK_TOP3 indicator while Models 3 and 4 use HH_BANKED to measure Bank (financial inclusion). Models 1 and 3 measure economic activity using sum of stable lights and Models 2 and 4 using lit pixels in the nightlights data.

Our results indicate that climate risk is strongly and positively associated with the fraction of liquid assets. Climate risk one-standard-deviation above the sample mean is associated with 5–12 percentage points larger liquid asset component in total assets of rural households (Fig. 2, Table S3). This is a substantial effect given that, on average, a household holds 15.6% of its assets in liquid form. However, access to formal financial institutions mitigates this effect to a large extent. Relative to the unbanked, the financially included hold 1–13 percentage points fewer assets in liquid form, when exposed to one-standard-deviation higher climate risk (Fig. 2, Table S3). We draw similar conclusions about the effect of climate risk on the banked and unbanked using several other specifications (Tables S4 and S5).

Taking these results together, we interpret that households respond to higher climate risk by keeping a larger buffer in the form of liquid assets. In resource-poor settings characterized by small landholdings and limited diversification opportunities, this reduces the resources available to households to make productive investments and/or increase consumption. However, financial inclusion alleviates the need to hold liquidity, relieving the resource constraint and enabling rural households to respond better to known climate risks.

**Figure 3:**
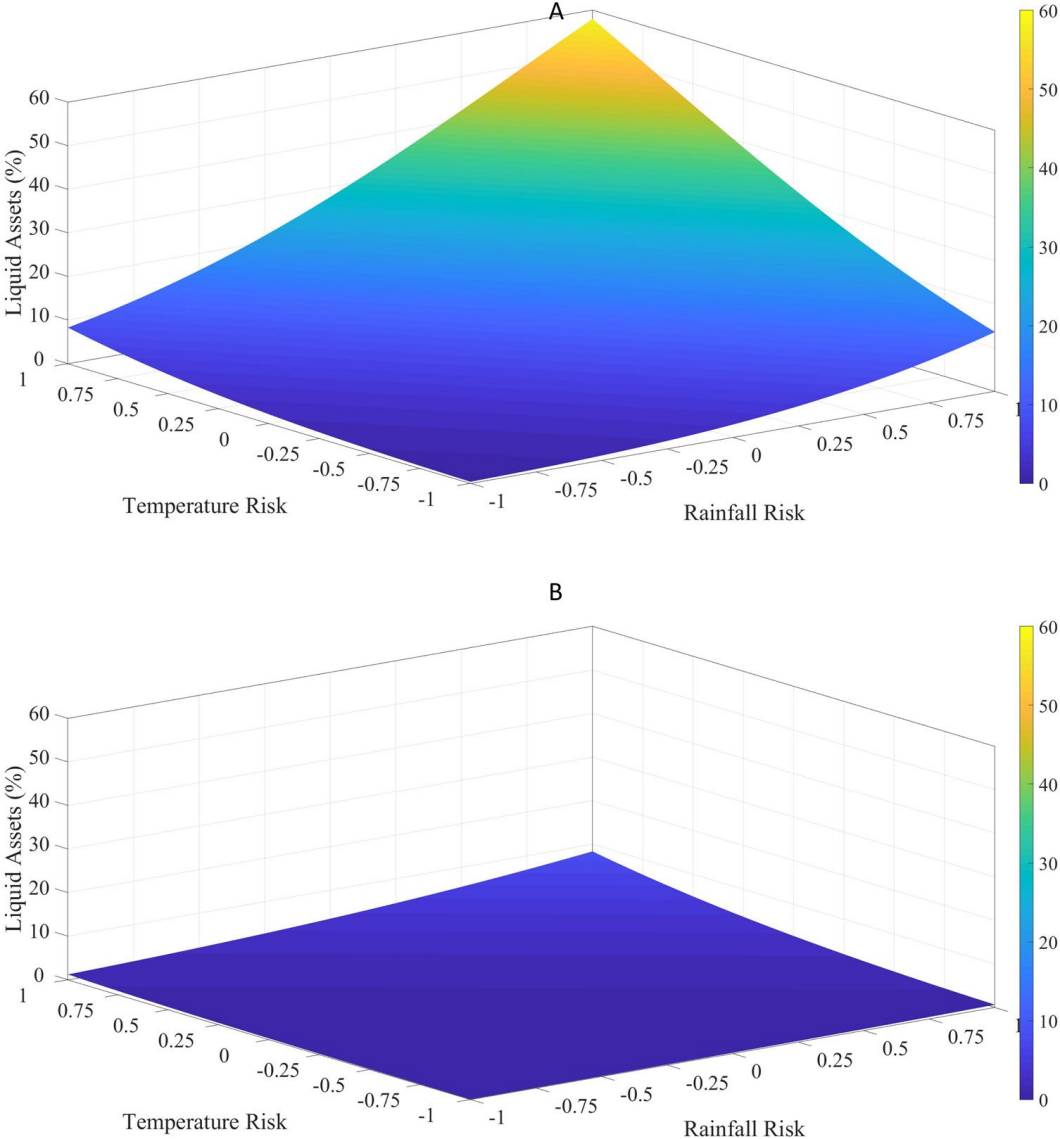
This figure shows the predicted percentage of liquid assets using results from Model 2 in Fig. 2 for (**A**) the financially excluded households (BANK = 0) and (**B**) the financially included households (BANK = 1). Temperature Risk and Climate Risk varies from -1 to + 1 standard deviation. All other explanatory variables are at their mean.

To better understand the extent to which financial inclusion frees up resources, we examine predicted values of percentage of liquid assets for the banked and unbanked for varying degrees of climate risk. When rainfall risk and temperature risk are both one-standard-deviation above their mean, the unbanked are predicted to hold nearly 50% in liquid assets (Fig. 3A). In such a high-risk scenario, the banked are projected to keep less than 20% liquidity in their asset portfolio (Fig. 3B). These results imply a sizeable effect of financial inclusion, given that current models of climate change predict an increase in both temperature and rainfall risk over the next few decades. In regions facing high climate risk, financial inclusion will reduce the resources that households need to keep in liquid form and therefore make them available for productive investments to address climate risk.

**Figure 4:**
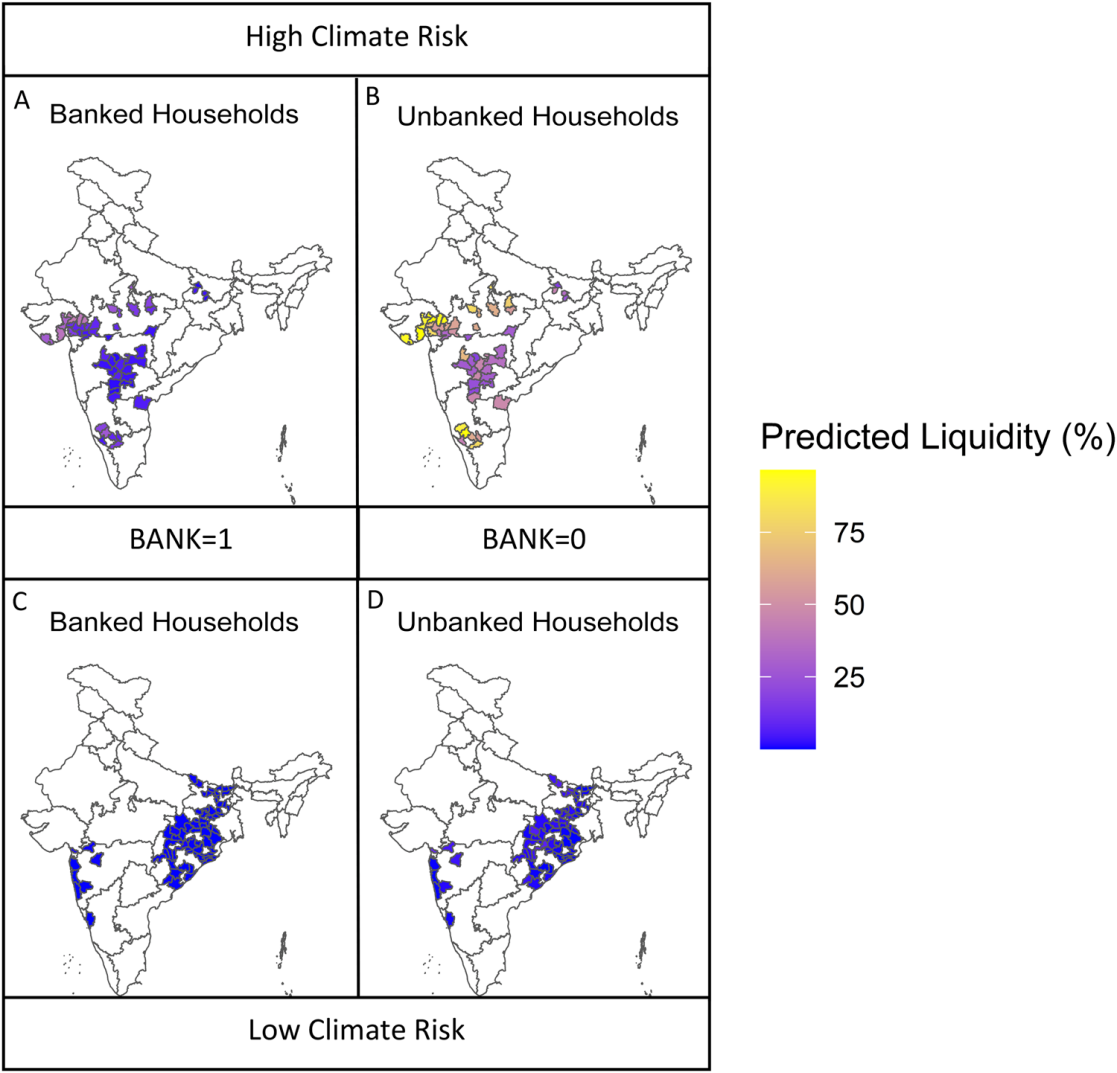
This figure shows the predicted liquid asset (%) for high (**A** and **B**) and low climate risk (**C** and **D**) districts in the range of VDSA climate risk variables in the 12 Indian states in the semi-arid tropics from the NSSO Debt and Investments survey. Predictions use the coefficients for Bank, Rainfall Risk, Temperature Risk, and their interaction with Bank from Model 2 in Fig. 2. Rainfall Risk and Temperature Risk are district-level variables standardized across all districts. Bank = 0 for financially included households (**B** and **D**) and 1 for financially excluded households (**A** and **C**). All other explanatory variables are at their VDSA sample mean. High (Low) climate risk districts have Rainfall and Temperature Risk above (below) their respective medians across districts. Map was produced using R statistical software (packages ‘rgdal’, ‘dplyr’, ‘sf’, and ‘ggplot2)’. Base maps were accessed from Bharat Maps (https://bharatmaps.gov.in/), the Geospatial Technology and Services Division of the Ministry of Electronics and Information Technology, Government of India.

To understand the implications for our findings beyond the 30 villages in our sample, we examine all the districts in 12 Indian states in the semi-arid tropics from the NSSO Debt and Investments survey. For each district, we calculate temperature risk and rainfall risk in the same way we calculate it for our sample villages. While the distribution of climate risk is similar for these larger sample of districts (Figs. S5B and S5C), we restrict the sample to the districts that fall within the range of VDSA climate risk variables. We estimate the percent of assets held in liquid form for banked and unbanked households using our main results (Fig. 2, Model 2). As we move from districts with low climate risk to those with high climate risk, we see a steep rise in the predicted liquidity ratio of unbanked households (Fig. 4B,D). The same comparison for banked households (Fig. 4A,C) shows a significantly less pronounced increase in liquid assets. These results further highlight the role financial inclusion can play in helping rural households respond to climate change by enabling them to reduce their unproductive liquid assets held as a buffer in response to climate risk.

Our results have important implications for the broader discussion about policy actions to enable adaptation to changing and increasing climate risk. Current knowledge of impacts of climate change is severely restricted by the coarse resolution of general circulation models. Even where scientists are confident about their projections regarding changing exposure to climate risks, policymakers still need to consider the differential vulnerability of affected populations in designing effective climate adaptation policies. Rural households in the semi-arid tropics are particularly sensitive to climate variability due to the nature of their livelihoods. They have adapted to climate risk by keeping a high proportion of their assets in forms amenable to relatively quick liquidation in response to income shocks or crises.

Given the differential vulnerability of rural households to climate change, specific climate actions are unlikely to benefit all households. For example, subsidies to encourage use of green manure can increase the ability of soils to retain moisture, enabling crops to withstand longer and harsher dry spells. But such an intervention will only help farmers that grow the crops that are at risk of dry spells. It will not benefit farmers growing drought-resistant varieties, or households with non-crop livelihood practices such as wage labor, wild collection, or service provision. These rural households, though affected by droughts indirectly through their dependence on farmers, will likely continue to use their liquid assets to meet expenses during droughts.

Financial inclusion puts resources directly in the hands of rural households in the form of relief from holding liquid assets, and this benefit increases with climate risks. Such a decentralized benefit also allows each household to reallocate resources to uses that are most appropriate to and closely aligned with specific livelihood portfolios. The cumulative economic benefit of a distributed pattern of investments will far outweigh anything that a central planner can accomplish with public investments in adaptation, especially given the lack of granular information regarding risks and opportunities at the household level^12^. Known benefits of financial services to mitigate climate risk, such as insurance, cheap credit, and long-term savings, will also only accrue after rural households are able to access formal institutions in the financial services sector. Our results show that expanding financial inclusion for the poor has clear climate adaptation benefits for rural households in semi-arid tropics facing high climate risks.

## Supplementary Information

Supplementary Information.

## Data Availability

All data and code necessary to reproduce results reported here are available at https://data.mendeley.com/datasets/rmsjdwht28.

## References

[1] Owen G (2020). What makes climate change adaptation effective? A systematic review of the literature. Glob. Environ. Chang..

[2] Chausson A, Turner B, Seddon D, Chabaneix N, Girardin CAJ, Kapos V, Key I, Roe D, Smith A, Woroniecki S, Seddon N (2020). Mapping the effectiveness of nature-based solutions for climate change adaptation. Glob. Chang. Biol..

[3] Janssens C, Havlík P, Krisztin T, Baker J, Frank S, Hasegawa T, Leclère D, Ohrel S, Ragnauth S, Schmid E, Valin H, Van Lipzig N, Maertens M (2020). Global hunger and climate change adaptation through international trade. Nat. Clim. Chang..

[4] Brown PR, Afroz S, Chialue L, Chiranjeevi T, El S, Grünbühel CM, Khan I, Pitkin C, Reddy VR, Roth CH, Sacklokham S, Williams LJ (2019). Constraints to the capacity of smallholder farming households to adapt to climate change in South and Southeast Asia. Clim. Dev..

[5] Ellis F (1998). Household strategies and rural livelihood diversification. J. Dev. Stud..

[6] Asfaw S, Scognamillo A, Di Caprera G, Sitko N, Ignaciuk A (2019). Heterogeneous impact of livelihood diversification on household welfare: Cross-country evidence from Sub-Saharan Africa. World Dev..

[7] Anderson SC, Ward EJ, Shelton AO, Adkison MD, Beaudreau AH, Brenner RE, Haynie AC, Shriver JC, Watson JT, Williams BC (2017). Benefits and risks of diversification for individual fishers. Proc. Natl. Acad. Sci..

[8] Andrijevic M, Crespo Cuaresma J, Muttarak R, Schleussner CF (2020). Governance in socioeconomic pathways and its role for future adaptive capacity. Nat. Sustain..

[9] Hansen J, Hellin J, Rosenstock T, Fisher E, Cairns J, Stirling C, Lamanna C, van Etten J, Rose A, Campbell B (2019). Climate risk management and rural poverty reduction. Agric. Syst..

[10] Kirkby P, Williams C, Huq S (2017). Climate and Development Community-based adaptation (CBA): adding conceptual clarity to the approach, and establishing its principles and challenges Community-based adaptation (CBA): Adding conceptual clarity to the approach, and establishing its principle. Taylor Fr..

[11] Sovacool BK, Linnér BO, Klein RJT (2017). Climate change adaptation and the least developed countries fund (LDCF): Qualitative insights from policy implementation in the Asia-Pacific. Clim. Change..

[12] Hill AC (2021). COVID’s lesson for climate research: Go local. Nature.

[13] C. B. Barrett, in *Agricultural Economics*, vol. 32, pp. 45–60 (2005).

[14] Fafchamps M, Lund S (2003). Risk-sharing networks in rural Philippines. J. Dev. Econ..

[15] Banerjee, A. Duflo, E. *Poor economics: Rethinking poverty and the ways to end it.* Public Affairs, Boston (2011).

[16] Wunder S, Noack F, Angelsen A (2018). Climate, crops, and forests: A pan-tropical analysis of household income generation. Environ. Dev. Econ..

[17] Deaton A (1989). Saving in developing countries: Theory and review. World Bank Econ. Rev..

[18] Rosenzweig MR, Wolpin KI (1993). Credit market constraints, consumption smoothing, and the accumulation of durable production assets in low-income countries: Investments in bullocks in India. J. Polit. Econ..

[19] Lee JJ, Sawada Y (2010). Precautionary saving under liquidity constraints: Evidence from rural Pakistan. J. Dev. Econ..

[20] Demirguc-kunt, A., Klapper, L. & Singer, D., Financial inclusion and inclusive growth: A review of recent empirical evidence. *World Bank Policy Resarch Working Paper* (8040, 2017).

[21] Karlan D, Osei R, Osei-Akoto I, Udry C (2014). Agricultural decisions after relaxing credit and risk constraints. Q. J. Econ..

[22] Cole S, Giné X, Vickery J (2017). How does risk management influence production decisions? Evidence from a field experiment. Rev. Financ. Stud..

[23] Carroll CD, Holm MB, Kimball MS (2021). Liquidity constraints and precautionary saving. J. Econ. Theory..

[24] GV, A. & Falk, T., Data on primary survey study on agricultural productivity and plot size: Village Dynamics in South Asia (VDSA), ICRISAT Dataverse, V1 (2020).

[25] Camara, N. & Tuesta, D., Measuring financial inclusion: A muldimensional index. BBVA Research Paper, (14/26) (2014).

[26] Avom D, Bangaké C, Ndoya H (2021). Measuring financial inclusion in African countries. Econ. Bull..

[27] Burgess R, Pande R (2005). Do rural banks matter? Evidence from the Indian social banking experiment. Am. Econ. Rev..

[28] Panagariya, A., Bank branch expansion and poverty reduction: A comment. Working Paper, Columbia University (2006).

[29] Kochar A (2011). The distributive consequences of social banking: A microempirical analysis of the Indian experience. Econ. Dev. Cult. Change..

[30] Garg, S., Gupta, S. & Mallick, S., Financial access and gender-wise entrepreneurship: Evidence from rural India. Working Paper (2022).

[31] Narayanan S (2016). The productivity of agricultural credit in India. Agric. Econ..

[32] Young, N., Banking and growth: Evidence from a regression discontinuity analysis. EBRD Working Paper No. 207 (2017).

[33] Pai DS, Sridhar L, Rajeevan M, Sreejith OP, Satbhai NS, Mukhopadhyay B (2014). Development of a new high spatial resolution (0.25° X 0.25°) Long period (1901–2010) daily gridded rainfall data set over India and its comparison with existing data sets over the region. Mausam.

[34] Srivastava AK, Rajeevan M, Kshirsagar SR (2009). Development of a high resolution daily gridded temperature data set (1969–2005) for the Indian region. Atmos. Sci. Lett..

[35] Office of the Registrar General and Census Commissioner of the Ministry of Home Affairs of the Government of India, Census of India (2011). https://censusindia.gov.in/DigitalLibrary/Tables.aspx.

[36] Defence Meteorological Satellites Program—Operational Linescan System (DMSP-OLS) night time lights (NTLs) dataset. Image and Data processing by NOAA's National Geophysical Data Center. DMSP data collected by the US Air Force Weather Agency. https://ngdc.noaa.gov/eog/dmsp/downloadV4composites.html.

[37] National Sample Survey Office at the Ministry of Statistics and Programme Implementation of the Government of India, of India, India - Debt and Investment Visit 2 - NSS 70th Round (2013). http://microdata.gov.in/nada43/index.php/catalog/132/related_materials.

[38] Ackerberg DA, Botticini M (2002). Endogenous matching and the empirical determinants of contract form. J. Polit. Econ..

[39] Bottazzi L, Da Rin M, Hellmann T (2008). Who are the active investors?: Evidence from venture capital. J. Financ. Econ..

[40] Tian X (2011). The causes and consequences of venture capital stage financing. J. Financ. Econ..

[41] Brander JA, Du Q, Hellman T (2015). The effects of government-sponsored venture capital: International evidence. Rev Financ..

[42] Wooldridge JM (2002). Econometric Analysis of Cross Section and Panel Data.

