# Supplementary material for: Financial inclusion helps rural households address climate risk
Source: Sci Rep. 2023 May 16;13:7929. doi: 10.1038/s41598-023-34844-y (PMC10188445; doi:10.1038/s41598-023-34844-y)
Supplement: Supplementary file 1 — Supplementary Information. [file 41598_2023_34844_MOESM1_ESM.pdf]

Supplementary Materials for

Financial Inclusion Helps Rural Households Address Climate Risk

Ashwini Chhatre   Prachi Deuskar   Javed Mohib   Deepanshi Bhardwaj

Correspondence to: ashwini_chhatre@isb.edu

This PDF file includes:

Materials and Methods
Figs. S1 to S5
Tables S1 to S5
Data S1

Materials and Methods

ICRISAT-VDSA Household Dataset

The International Crops Research Institute for the Semi-Arid Tropics (ICRISAT) conducts village level studies to track rural poverty in households in village economies in South Asia under the Village Dynamics studies in South Asia (VDSA) initiative (24). The ICRISAT-VDSA dataset provides granular household-level data about resource endowments, cultivation input and output, employment, livestock, cropping pattern, financial transactions, and development program participation. It also has information about the demographics of each household such as caste, landholding, and household size. The presence of a strong panel component, temporal granularity of data, village-level household identification, high quality of refinement, and the ability to track households across 5 years makes it a suitable dataset for our study. We excluded households that were not present in the data for all five years as well as households that were subsequently included as replacements. We have a balanced panel of annual data for 1082 households spanning across 30 villages in semi-arid tropics (states of Andhra Pradesh, Maharashtra, Madhya Pradesh, Gujarat, Bihar, Jharkhand, and Orissa in India).

The VDSA survey includes the following two questions for all households: (1) Did you experience any severe drought/flood/pest/ diseases that affected your livelihoods during the last year? (2) Conditional on experiencing a problem, list the coping mechanisms adopted in order of importance. The distribution of households facing climate shocks over the 5-year period (Fig. S1) shows that these rural households face substantial climate risk. 59% households experienced climate shocks – drought, flood, cyclone, heavy rainfall, or frost – in at least one year. 13% of households experienced climate shocks in more than 2 of the 5 years. Out of the 633 households who have faced climate shock at least once, for a majority (57%) the primary coping mechanism is their own savings (Fig. S2). Given dependence on own savings in the face of climate risk, our main variable of interest is the proportion of liquid assets held by the households. We define the primary variables used in this study in Table S1.

A follow up question on coping mechanism in the survey asks households to rank the individuals/ institutions they approach for assistance based on the reliability of these sources in the event of a drought or a flood. The geography of our study being a semi-arid region, the households are much more likely to face drought: out of 633 households that reported facing climate shocks, 514 households have experienced drought at least once in our sample. Thus, we focus on reliable sources of assistance in the event of drought. 'Kin and relatives' and 'Friends' are the top 2 reliable sources of assistance for the households in our sample. (Fig. S3). Bank, the 5th most common source of assistance, features in the top 3 reliable sources for 552 households. When households can rely on formal financial institutions, they rely on the moneylender less (Fig. S4). We create a variable BANK_TOP3 equal to 1 if a household in a year reports bank in its top three reliable sources of assistance. This is one of our indicators of financial inclusion. The second variable, HH_BANKED is 1 if the households borrow from or save with a bank or a formal financial institution. Table S2 reports the descriptive statistics of all our main variables.

Climate Variability

For creating climate risk variables, we use rainfall and temperature variables from India Meteorological Department (IMD) Data from 1951-2014, published by the Ministry of Earth Science, Government of India (33-34). Since our sample consists of rural households primarily dependent agriculture, we focus on the climate variability during monsoon, the primary growing season in India. For each village using annual data from 1951 to 2014 we calculate the coefficient of variation of total rainfall and mean daily temperature for the 120 days following the onset of the monsoon.

Village Infrastructure and Economic Activity

In addition to the household and climate variables, we use infrastructure and economic activity indicators at the village level as instruments or controls in our estimation. Based on 2001 Village Census (35), we create indicators whether the village has a paved road or a power connection within 5 km. To control for economic activity, we use the Defence Meteorological Satellites Program – Operational Linescan System (DMSP-OLS) night time lights (NTLs) dataset (36) to create village-level night lights variables as measured in 2010.

Comparison: Our Sample and Broader Sample of Rural Households

We compare the distribution of our key variables to a broader sample NSSO Debt and Investments Survey conducted in 2013 (37). We consider all the districts in the NSSO sample in 12 Indian states in the semi-arid tropical geography (Andhra Pradesh, Bihar, Chhattisgarh, Goa, Gujarat, Jharkhand, Karnataka, Madhya Pradesh, Maharashtra, Odisha, Rajasthan, and Telangana). We restrict our sample to households who have 30% or less land irrigated to make our sample comparable to the VDSA sample. Fig S5 shows the distribution of Liquidity Ratio (%) and the climate risk variables for the VDSA and the NSSO samples. The distributions of the variables across the two samples are generally similar, indicating that the VDSA sample is representative of the broader sample of rural Indian households.

Estimation Approach

We want to investigate how proportion of saved assets responds to climate risk, financial inclusion, and the interaction of the two. However, there are two potential problems in drawing causal inference based on an OLS regression. First, the household's participation in the formal modes of financial savings and credit can depend on many of its observed and unobserved characteristics. While we control for relevant observed ones (including fixed effects for household landholding and caste), we cannot control for the unobserved attributes. Second, there is the possibility of reverse causality – households with a certain level of desired liquid assets may choose to access financial institutions. Hence, high proportion of liquid assets may drive financial inclusion instead of (or in addition

to) financial inclusion influencing the level of liquidity. To address these problems, we use an instrumental variable regression. Our objective in conducting an instrumental variable regression is capture “supply” of formal financial institutions by a variable that is uncorrelated with “demand” at the household level. Following the approach suggested by (38), applied (39-41), we use village indicators as our instrument. The argument is that whether a household is financially included by a bank depends on the relevant characteristics of the household as well as the bank. The inclusion also depends on distribution of households and proximate bank branches in each village. (38) argue that this distribution is exogenous and recommend use of a set of geography-based instruments. In order to satisfy the exclusion restriction, appropriate instruments for our regression specifications should affect the selection into financial inclusion but should not directly be related to the outcome (household liquidity) after adding the relevant control variables.

One concern is using village indicators as instruments is that some village level characteristics in particular economic prosperity and climate risk might affect financial inclusion and also be directly related to level of household liquidity. We address this concern by including village night lights and climate risk variables as controls in our second stage regression. Additionally, we include district fixed effects to absorb unobserved characteristics such as district-level economic activity that may not be fully captured by night lights.

We follow Procedure 18.1 for a binary endogenous variable, described in Chapter 18.4 of (42). Specifically, we run a probit regression of Bank, the financial inclusion indicator, as a function of village indicators and all the other explanatory variables (climate risk and controls) and calculate the predicted value BANK_HAT. Then we run an instrumental variable regression with INVNORM_LIQASSETS as the dependent variable and BANK_HAT, BANK_HAT*RAINFALL RISK, BANK_HAT*TEMPERATURE RISK as instruments for Bank, Bank*Rainfall Risk, Bank*Temperature Risk. INVNORM_LIQASSETS is the inverse normal transformation of the liquid assets fraction. We use this transformation so that any predicted value when converted back to liquid assets fraction will always lie between 0 and 1. Specifically, our estimation equations look as follows:

<u>Probit:</u> $P(BANK_{hvy} = 1) = \Phi(\alpha_0 + \alpha_{1v} I_v + \alpha_2 RAINFALL\ RISK_v + \alpha_3 TEMPERATURE\ RISK_v + Controls$

$BANK\_HAT_{hvy}$ = Fitted value of $BANK_{hvy}$ from the probit estimation.

First stage:

$$BANK_{hvy} = \beta_0 + \beta_1 BANK\_HAT_{hvy} + \beta_2 BANK\_HAT_{hvy} * RAINFALL\ RISK_v + \beta_3 BANK\_HAT_{hvy} * TEMPERATURE\ RISK_v + \beta_4 RAINFALL\ RISK_v + \beta_5 TEMPERATURE\ RISK_v + Controls$$

$$BANK_{hvy} * RAINFALL\ RISK_v = \gamma_0 + \gamma_1 BANK\_HAT_{hvy} + \gamma_2 BANK\_HAT_{hvy} * RAINFALL\ RISK_v + \gamma_3 BANK\_HAT_{hvy} * TEMPERATURE\ RISK_v + \gamma_4 RAINFALL\ RISK_v + \gamma_5 TEMPERATURE\ RISK_v + Controls$$

$$BANK_{hvy} * TEMPERATURE\ RISK_v = \delta_0 + \beta_1 BANK\_HAT_{hvy} + \delta_2 BANK\_HAT_{hvy} * RAINFALL\ RISK_v + \delta_3 BANK\_HAT_{hvy} * TEMPERATURE\ RISK_v + \delta_4 RAINFALL\ RISK_v + \delta_5 TEMPERATURE\ RISK_v + Controls$$

$\widehat{BANK}_{hvy}, BANK_{hvy} * \widehat{RAINFALL}\ RISK_v, BANK\_HAT_{hvy} * \widehat{TEMPERATURE}\ RISK_v$ are fitted values from the first stage.

<u>Second stage:</u>

$$INVNORM\_LIQASSETS_{\ hvy} = \lambda_0 + \lambda_1 \widehat{BANK}_{hvy} + \lambda_2 BANK_{hvy} * \widehat{RAINFALL}\ RISK_v + \lambda_3 BANK_{hvy} * \widehat{TEMPERATURE}\ RISK_v + \lambda_4 RAINFALL\ RISK_v + \lambda_5 TEMPERATURE\ RISK_v + Controls\ ,$$

where $BANK_{hvy}$ is 1 if household h, from village v, in year y is financially included and 0 otherwise. I_v are village indicator variables. Controls include HOUSEHOLD SIZE, VILLAGE ALTITUDE, RAINFALL RISK, TEMPERATURE RISK, NIGHTLIGHTS, and district, caste and class fixed effects.

Table S3 presents the results for the second stage of our main specifications, which use HH_BANKED or BANK_TOP3 as Bank, the financial inclusion indicator and control for night lights using either SUM OF STABLE LIGHTS or LIT PIXELS. In all the four specifications in Table S3, the Kleibergen-Paap Wald F statistic for weak instruments ranges from 51.17 to 71.73 indicating that the instruments are strong. The effect of both the climate risk variables on liquid assets is positive and statistically significant, indicating that households facing greater climate risk hold a larger fraction in liquid assets as a precaution. Further, coefficients for the interaction of financial inclusion and RAINFALL RISK variables are negative and significant for all models. While the interaction of TEMPERATURE RISK and BANK is insignificant in Table S3, it is negative and mostly significant in the alternative specifications discussed below. Thus, we find that financial inclusion mitigates the need to hold liquid assets as a coping mechanism for climate risk.

Table S4 presents the first stage results of the models in Table S3. We see that pseudo R-squared for the probit model ranges from 0.236 to 0.269, providing support, in addition to the F-statistic reported in Table S3, for the strength of the instrument. Further, we see that in the first stage regressions the coefficient for BANK_HAT, BANK_HAT*RAINFALL RISK, and BANK_HAT*TEMPERATURE RISK in regression equations of BANK, BANK*RAINFALL RISK, and BANK*TEMPERATURE RISK, respectively, is positive, highly significant and close to 1, indicating the validity of the instruments.

Alternative Specifications

To investigate robustness of results we estimate various alternative specifications. We use one climate risk variable at a time (Table S5). We use OLS instead of instrumental variable (Table S^) and within OLS we include village fixed

effects (Columns 3-4 of Table S6). All these results broadly support the conclusions that i) households hold more liquid assets when facing greater climate risk ii) financial inclusion reduces the need to hold liquid assets in response to climate risk.

We note that the coefficient of the BANK variable is positive and significant in the OLS specification (Table S6). This is line with the pattern in Figure 1D. However, the coefficient is negative and significant when we use the instrumental variable approach (Tables S3 and S5). Thus, the positive relationship observed in Figure 1D and Table S6 is likely to be due to households with greater need or ability to save choosing to be banked. Once we control for this household-level selection through instrumental variables, we see that financial inclusion results in lower liquidity ratio, in line with our expectation.

References and Notes

24. A. GV, T. Falk, Data on primary survey study on agricultural productivity and plot size: Village Dynamics in South Asia (VDSA), ICRISAT Dataverse, V1 (2020).

33. D. S. Pai, L. Sridhar, M. Rajeevan, O. P. Sreejith, N. S. Satbhai, B. Mukhopadhyay, Development of a new high spatial resolution (0.25° X 0.25°) Long period (1901-2010) daily gridded rainfall data set over India and its comparison with existing data sets over the region. *MAUSAM*. **65**, 1–18 (2014).

34. A. K. Srivastava, M. Rajeevan, S. R. Kshirsagar, Development of a high resolution daily gridded temperature data set (1969-2005) for the Indian region. Atmos. Sci. Lett. 10, 249–254 (2009).

35. Office of the Registrar General and Census Commissioner of the Ministry of Home Affairs of the Government of India, Census of India (2011). https://censusindia.gov.in/DigitalLibrary/Tables.aspx

36. Defence Meteorological Satellites Program – Operational Linescan System (DMSP-OLS) night time lights (NTLs) dataset. Image and Data processing by NOAA's National Geophysical Data Center. DMSP data collected by the US Air Force Weather Agency. https://ngdc.noaa.gov/eog/dmsp/downloadV4composites.html

37. National Sample Survey Office at the Ministry of Statistics and Programme Implementation of the Government of India, of India, India - Debt and Investment Visit 2 - NSS 70th Round (2013). http://microdata.gov.in/nada43/index.php/catalog/132/related_materials

38. D.A. Ackerberg, M. Botticini, Endogenous matching and the empirical determinants of contract form. *J. Political Econ.* **110**, 564-591 (2002).

39. L. Bottazzi, M. Da Rin, T. Hellmann, Who are the active investors?: Evidence from venture capital. *J. Financ. Econ*. **89**, 488-512 (2008).

40. X. Tian, The causes and consequences of venture capital stage financing. *J. Financ. Econ.* **101**, 132-159 (2011).

41. J. A. Brander, Q. Du, T. Hellman, The Effects of Government-Sponsored Venture Capital: International Evidence. *Rev Financ.* **19**, 571-618 (2015).

42. J. M. Wooldridge, Econometric Analysis of Cross Section and Panel Data (The MIT Press, Cambridge, Massachusetts, 2002).

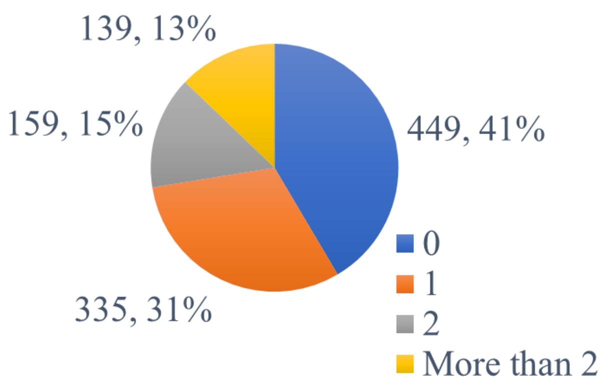

Fig. S1. Households by number of years in which they faced adverse climate shocks. Adverse climate shocks include drought, flood, cyclone, heavy rainfall, and frost.

Type or paste caption here. Create a page break and paste in the Figure above the caption.

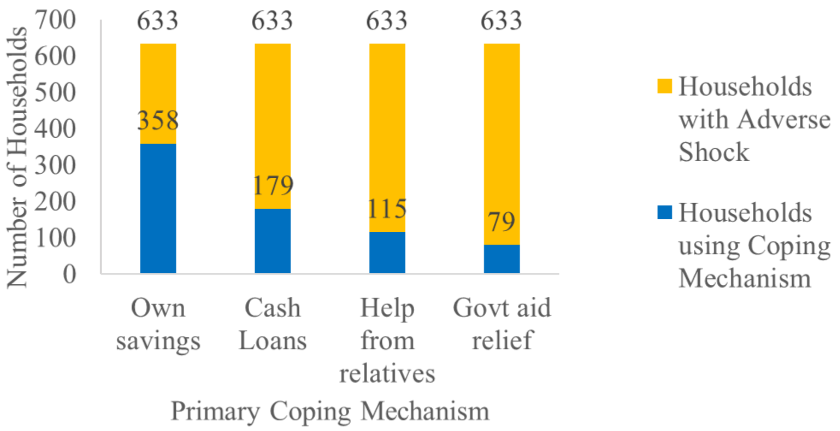

Fig. S2. Primary coping mechanism of households who have faced an adverse climate shock. The yellow bars show the total number of households who have faced an adverse climate shock affecting their livelihoods at least once in our sample. The blue bars show the number of households facing such a shock who have indicated a particular mechanism as their primary coping mechanism at least once.

Type or paste caption here. Create a page break and paste in the Figure above the caption.

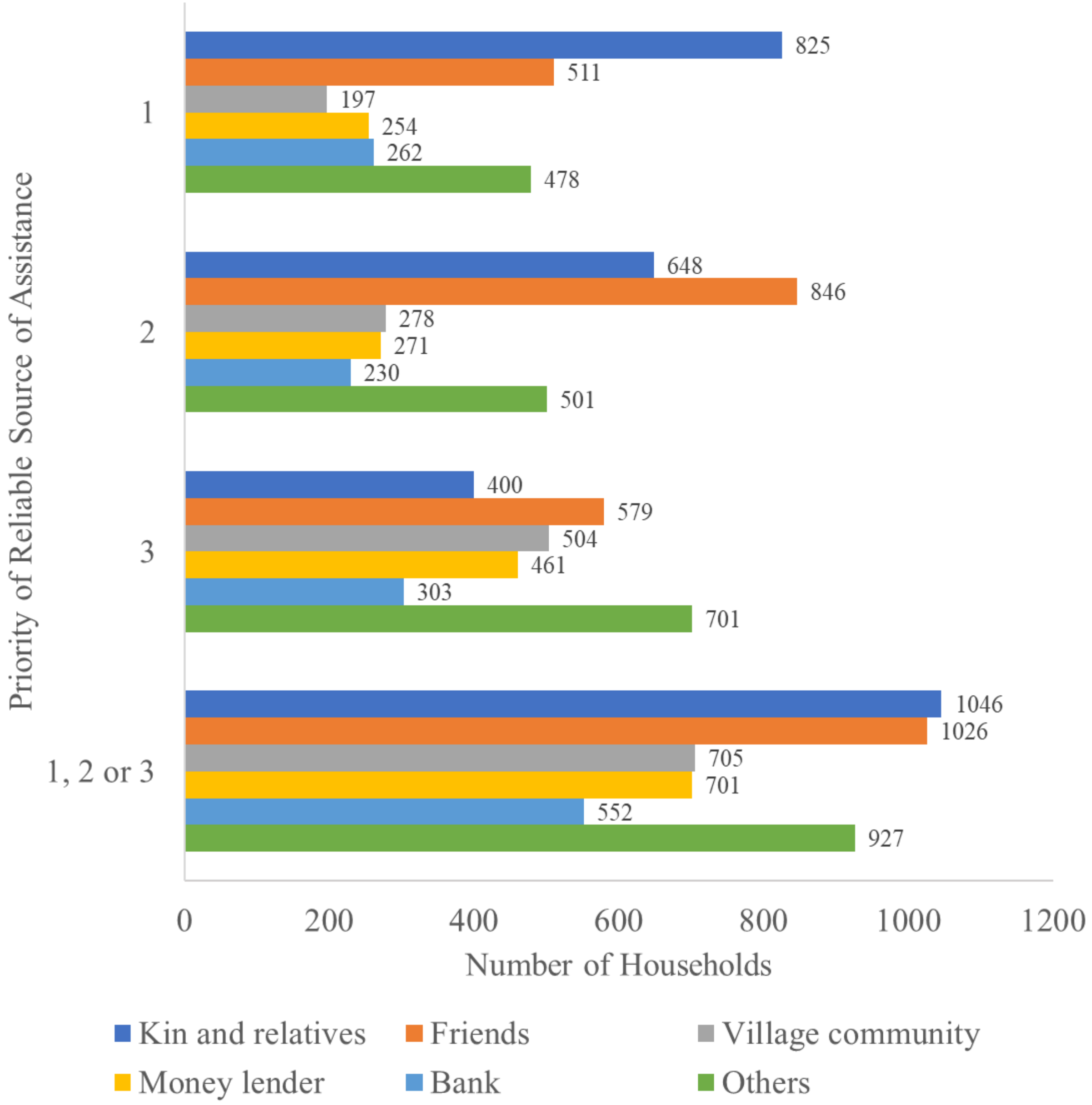

Fig. S3. Reliable sources of assistance in case of a drought. The bars show number of households who have at least once mentioned a particular source of assistance as their top, second, third or one of the top 3 reliable sources. "Others" include village local government, public distribution shops, self-help groups, local political groups, non-government organizations, and state or central government.

Type or paste caption here. Create a page break and paste in the Figure above the caption.

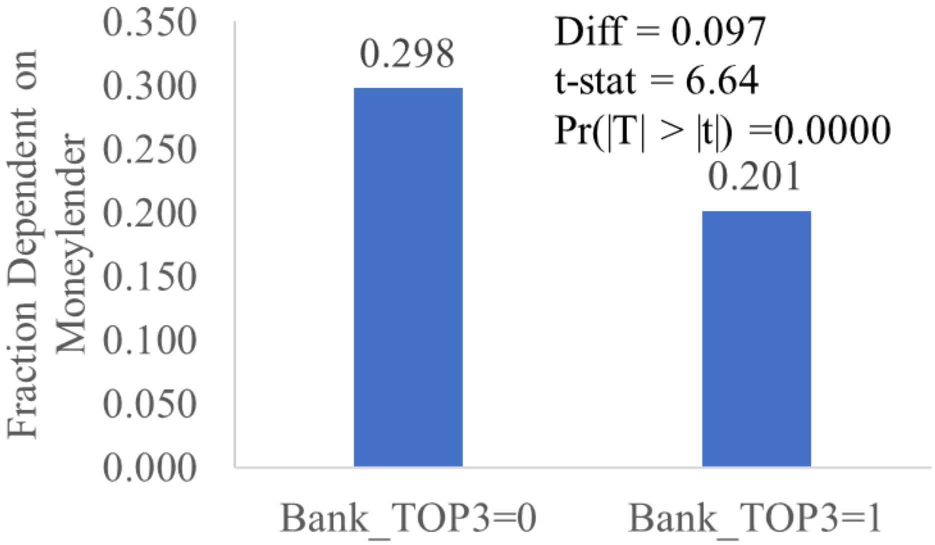

Fig. S4. Dependence of moneylender by reliability of bank. The bars show fraction of household-years where moneylender is in the top 3 reliable sources of assistance in case of a drought. Bank_TOP3 is an indicator equal to 1 if bank is in the top 3 reliable sources for a household for a year, and 0 otherwise.

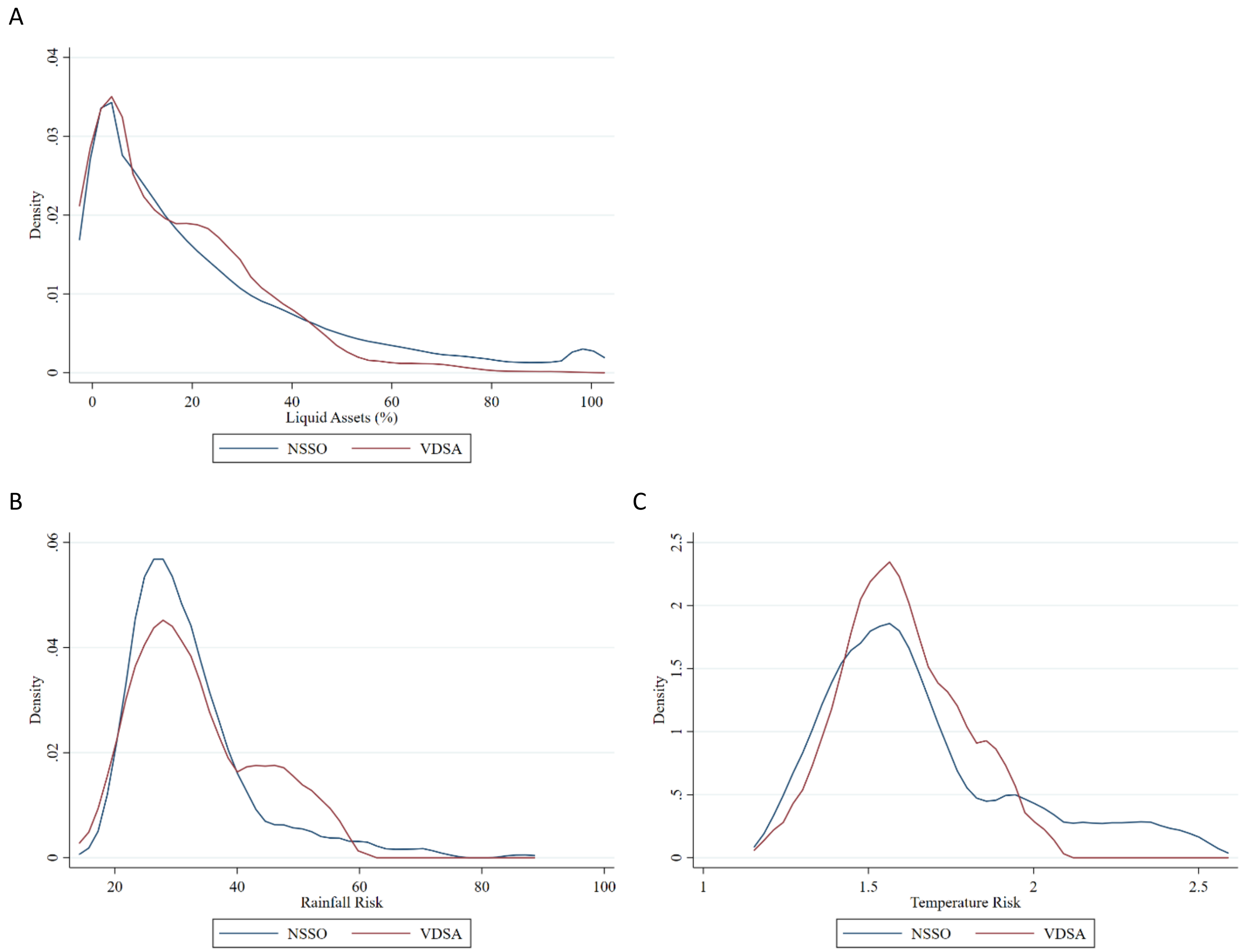

Fig. S5. Comparison of kernel density for the NSSO and the VDSA samples for percentage of liquid assets (A), rainfall risk (B) and temperature risk (C). Rainfall and temperature risk measured as the coefficient of variation of yearly rainfall and mean temperature from 1951 to 2014.

Variable	Definition
**Household-Year level variables**	
Dataset: ICRISAT-VDSA	
TOTAL ASSETS (INR)	Total assets in INR owned by a household. Sum of values of liquid assets, buildings, consumer durables, farm equipment, livestock, and inventory of livestock feed, fuel, farm inputs, and grain.
LIQUID ASSETS (INR)	Liquid assts in INR owned by a household. Sum of values of cash, gold and silver, savings in bank and other financial institutions, financial investments such as stocks and bonds.
LIQUID ASSETS (%)	$\frac{Liquid\ Assets\ (INR)}{Total\ Assets\ (INR)} x100$
INVNORM_LIQASSETS	Inverse normal transformation of $\frac{Liquid\ Assets\ (INR)}{Total\ Assets\ (INR)} + 0.0001$
BANK_TOP3	1 if the household has bank in its top 3 reliable sources of assistance in case of a drought, 0 otherwise.
HH_BANKED	1 if the household saves or borrows from banks or other formal financial intermediaries, 0 otherwise
HOUSEHOLD SIZE	Number of persons in the household
Farmer Class Indicators	Indicators for the following categories: Marginal (land holdings < 1 hectare), Small (land holdings between 1 and 2 hectares), Medium (land holdings between 2 and 10 hectares), Large (land holdings > 10 hectares)
Caste Indicators	Indicators for the following caste groups: (1) NT/SC/ST (2) BC/OBC/SBC/SEBC/EBC (3) FC
**Village-level variables**	
Dataset: IMD	
RAINFALL RISK	Coefficient of variation of yearly total rainfall at a village during 120 days after the monsoon onset from 1951 to 2014.
TEMPERATURE RISK	Coefficient of variation of yearly average daily mean temperature at a village during 120 days after the monsoon onset from 1951 to 2014.
Dataset: 2001 Village Census	
PAVED ROAD	1 if the village has a paved road, 0 otherwise.
POWER	1 if the village has grid electricity connection, 0 otherwise.
Dataset: DMSP-OLS Night Lights	
SUM OF STABLE LIGHTS	Sum of radiance values (ranging from 0 meaning no light to 63) of all the pixels within 5 km radius around village
LIT PIXELS	Number of radiant pixels within 5 km radius around village
Data source: Google Earth	
VILLAGE ALTITUDE (meters)	Elevation of the village from the sea level.

Table S1. Definition of the main variables.

Variable	Observations	Mean	Std Dev	Median	Interquartile Range
Dataset: ICRISAT-VDSA					
TOTAL ASSETS (INR)	5410	456,736	690,039	256,577	406,506
LIQUID ASSETS (INR)	5410	87,120	226,297	28,920	82,560
LIQUID ASSETS (%)	5410	15.56	15.72	11.3	22.5
INVNORM_LIQASSETS	5410	-1.53	1.17	-1.2	1.3
BANK_TOP3	5410	0.22	0.42	0	0
HH_BANKED	5410	0.45	0.5	0	1
HOUSEHOLD SIZE	5410	5.51	2.69	5	3
Dataset: IMD					
RAINFALL RISK	5410	33.75	9.51	29.9	14
TEMPERATURE RISK	5410	1.59	0.17	1.6	0.2
Dataset: 2001 Village Census					
PAVED ROAD	5410	0.64	0.48	1	1
POWER	5410	0.71	0.45	1	1
Dataset: DMSP Night Lights					
SUM OF STABLE LIGHTS	5410	395.13	330.05	424	516
LIT PIXELS	5410	49.78	33.9	72	71
Data source: Google Earth					
VILLAGE ALTITUDE (meters)	5410	276.92	229.5	205	394

Table S2. Descriptive statistics of the main variables. Each observation is a household-year. See Table S1 for variable definitions.

	Dependent variable = INVNORM_LIQASSETS			
BANK	-1.276***	-1.123***	-1.766***	-1.812***
	(0.185)	(0.175)	(0.220)	(0.229)
RAINFALL RISK	0.725***	0.795***	0.672***	0.770***
	(0.123)	(0.117)	(0.137)	(0.137)
RAINFALL RISK*BANK	-0.405***	-0.359***	-0.682***	-0.675***
	(0.129)	(0.127)	(0.130)	(0.132)
TEMPERATURE RISK	0.498***	0.649***	0.886***	1.003***
	(0.096)	(0.086)	(0.144)	(0.141)
TEMPERATURE RISK*BANK	-0.019	-0.101	0.169	0.185
	(0.116)	(0.110)	(0.122)	(0.123)
HOUSEHOLD SIZE	0.015***	0.014***	0.030***	0.030***
	(0.005)	(0.005)	(0.007)	(0.007)
VILLAGE ALTITUDE	-0.000	0.000	0.000	0.001**
	(0.000)	(0.000)	(0.000)	(0.000)
NIGHTLIGHTS	0.001***	0.007***	0.001***	0.007***
	(0.000)	(0.001)	(0.000)	(0.001)
Observations	5,410	5,410	5,410	5,410
Kleibergen-Paap Wald F statistic for weak instruments	57.65	51.17	71.73	71.19
Bank	BANK_TOP3	BANK_TOP3	HH_BANKED	HH_BANKED
Instrument	Village Indicators	Village Indicators	Village Indicators	Village Indicators
Fixed Effects	Class, Caste, District	Class, Caste, District	Class, Caste, District	Class, Caste, District
Nightlights	Sum of Stable Lights	Lit Pixels	Sum of Stable Lights	Lit Pixels

Table S3. Coefficient estimates from an instrumental variable regression of INVNORM_LIQASSETS on an indicator of financial inclusion (BANK), climate risk, and their interactions. See Table S1 for variable definitions. Rainfall and Temperature Risk are standardized to have 0 mean and unit standard deviation for the sample. Robust standard errors are in the parentheses. *, **, and *** indicate significance at 1%, 5%, and 10% respectively. The last 4 rows provide additional details of the specification, including whether Bank (financial inclusion) is measured using BANK_TOP3 or HH_BANKED.

First stage results. Second stage dependent variable = INVNORM_LIQASSETS				
		Probit		
Dependent variable	Bank	Bank	Bank	Bank
Pseudo R-squared	0.269	0.269	0.236	0.236
		First stage		
Dependent Variable 1	BANK	BANK	BANK	BANK
BANK_HAT	1.104***	1.109***	1.005***	1.006***
	(0.067)	(0.067)	(0.073)	(0.073)
RAINFALL RISK*BANK_HAT	-0.094**	-0.099**	-0.059	-0.059
	(0.045)	(0.046)	(0.039)	(0.039)
TEMPERATURE RISK*BANK_HAT	0.040	0.039	0.088**	0.088**
	(0.045)	(0.044)	(0.037)	(0.037)
Dependent Variable 2	RAINFALL RISK*BANK	RAINFALL RISK*BANK	RAINFALL RISK*BANK	RAINFALL RISK*BANK
BANK_HAT	0.045	0.050	0.025	0.027
	(0.073)	(0.069)	(0.080)	(0.084)
RAINFALL RISK*BANK_HAT	0.951***	0.948***	1.033***	1.033***
	(0.058)	(0.059)	(0.048)	(0.047)
TEMPERATURE RISK*BANK_HAT	-0.002	-0.004	-0.107***	-0.107***
	(0.043)	(0.041)	(0.033)	(0.033)
Dependent Variable 3	TEMPERATURE RISK*BANK	TEMPERATURE RISK*BANK	TEMPERATURE RISK*BANK	TEMPERATURE RISK*BANK
BANK_HAT	0.034	0.027	0.011	0.013
	(0.067)	(0.065)	(0.082)	(0.085)
RAINFALL RISK*BANK_HAT	-0.064	-0.064	-0.053	-0.053
	(0.040)	(0.041)	(0.038)	(0.038)
TEMPERATURE RISK*BANK_HAT	1.039***	1.043***	0.913***	0.912***
	(0.065)	(0.065)	(0.050)	(0.050)
		Additional Details		
Bank	BANK_TOP3	BANK_TOP3	HH_BANKED	HH_BANKED
Nightlights	Sum of Stable Lights	Lit Pixels	Sum of Stable Lights	Lit Pixels
Observations	5,410	5,410	5,410	5,410

Table S4. Results from the first stage of an instrumental variable regression of INVNORM_LIQASSETS on an indicator of financial inclusion (BANK), climate risk, and their interactions (Table S3). See Table S1 for variable definitions. Rainfall and Temperature Risk are standardized to have 0 mean and unit standard deviation for the sample. We follow the Procedure 18.1 for a binary endogenous variable, described in Chapter 18.4 of (30). Specifically, we run a probit regression of Bank, the financial inclusion indicator, as a function of village indicators and all the other explanatory variables (climate risk, controls, fixed effects) from the second stage and calculate the predicted value BANK_HAT. Then we run an instrumental variable regression with INVNORM_LIQASSETS as the dependent variable and BANK_HAT, BANK_HAT*RAINFALL RISK, BANK_HAT*TEMPERATURE RISK as instruments for Bank, Bank*Rainfall Risk, Bank*Temperature Risk. This table reports pseudo R-squared from the probit regression and selected coefficient estimates from the first stage regression. Robust standard errors are in the parentheses. *, **, and *** indicate significance at 1%, 5%, and 10% respectively. The last 3 rows provide additional details of the specification, including whether Bank (financial inclusion) is measured using BANK_TOP3 or HH_BANKED.

Dependent variable = INVNORM_LIQASSETS				
Bank	-1.116***	-0.803***	-1.132***	-1.420***
	(0.148)	(0.135)	(0.175)	(0.192)
Rainfall Risk	0.493***		0.168**	
	(0.092)		(0.079)	
Rainfall Risk*Bank	-0.277**		-0.215**	
	(0.126)		(0.086)	
Temperature Risk		0.315***		0.587***
		(0.061)		(0.089)
Temperature Risk*Bank		-0.406***		-0.156
		(0.101)		(0.112)
Household Size	0.014***	0.015***	0.024***	0.031***
	(0.005)	(0.005)	(0.006)	(0.006)
Village Altitude	0.000	0.001***	0.001**	0.001***
	(0.000)	(0.000)	(0.000)	(0.000)
Nightlights	0.009***	0.006***	0.008***	0.007***
	(0.001)	(0.001)	(0.001)	(0.001)
Observations	5,410	5,410	5,410	5,410
Kleibergen-Paap Wald F statistic for weak instruments	120.4	109.4	131.2	125.2
Bank	BANK_TOP3	BANK_TOP3	HH_BANKED	HH_BANKED
Instrument	Village Indicators	Village Indicators	Village Indicators	Village Indicators
Fixed Effects	Class, Caste, District	Class, Caste, District	Class, Caste, District	Class, Caste, District
Nightlights	Lit Pixels	Lit Pixels	Lit Pixels	Lit Pixels

Table S5. Coefficient estimates from an instrumental variable regression of INVNORM_LIQASSETS on an indicator of financial inclusion (BANK), climate risk, and their interactions. See Table S1 for variable definitions. Rainfall and Temperature Risk are standardized to have 0 mean and unit standard deviation for the sample. Robust standard errors are in the parentheses. *, **, and *** indicate significance at 1%, 5%, and 10% respectively. The last 5 rows provide additional details of the specification, including whether Bank (financial inclusion) is measured using BANK_TOP3 or HH_BANKED.

	Dependent variable = INVNORM_LIQASSETS			
BANK	0.179***	0.364***	0.256***	0.451***
	(0.031)	(0.028)	(0.030)	(0.027)
RAINFALL RISK	0.396***	0.444***		
	(0.062)	(0.060)		
RAINFALL RISK*BANK	-0.084***	-0.063**	-0.085***	-0.075***
	(0.030)	(0.029)	(0.028)	(0.028)
TEMPERATURE RISK	0.485***	0.468***		
	(0.067)	(0.066)		
TEMPERATURE RISK*BANK	-0.229***	-0.149***	-0.147***	-0.171***
	(0.034)	(0.029)	(0.032)	(0.027)
HOUSEHOLD SIZE	0.012***	0.009**	0.017***	0.014***
	(0.005)	(0.004)	(0.004)	(0.004)
VILLAGE ALTITUDE	0.001***	0.001***		
	(0.000)	(0.000)		
NIGHTLIGHTS	0.002***	0.002***		
	(0.001)	(0.001)		
Observations	5,410	5,410	5,410	5,410
R-squared	0.429	0.443	0.495	0.519
Bank	BANK_TOP3	HH_BANKED	BANK_TOP3	HH_BANKED
Estimation	OLS Regression	OLS Regression	OLS Regression	OLS Regression
Fixed Effects	Class, Caste, District	Class, Caste, District	Class, Caste, Village	Class, Caste, Village
Nightlights	Lit Pixels	Lit Pixels	Lit Pixels	Lit Pixels

Table S6. Coefficient estimates from an OLS regression of InvNorm_LiqAssets on an indicator of financial inclusion (Bank), climate risk, and their interactions. See Table S1 for variable definitions. Rainfall and Temperature Risk are standardized to have 0 mean and unit standard deviation for the sample Robust standard errors are in the parentheses. *, **, and *** indicate significance at 1%, 5%, and 10% respectively. The last 4 rows provide additional details of the specification, including whether Bank (financial inclusion) is measured using BANK_TOP3 or HH_BANKED.

Data S1. All data and code necessary to reproduce results reported here are available at https://data.mendeley.com/datasets/rmsjdwht28
